# 

**Published:** 1959-10

**Authors:** 


MEDICAL JOURNAL OF THE SOUTH-WEST

*(Incorporating the Bristol Medico-Chirurgical Journal)*

Established 1883

VOL. 74 (iv) October 1959 NO. 274

### *Contents*

BABY FARMING IN ENGLAND . . . . *Philip Evans* 83

THE USE OF AN ANIMAL LUNG AS OXYGENATOR IN A HEART-LUNG MACHINE . . *M. G. Wilson and others* 90

VOL. 75 (i) January 1960 NO. 275

CARDIAC ISCHAEMIA . . . . . *Dr. G. Mather* 1

A PATHOLOGIST'S SURVEY OF SUPERFICIAL TUMORS REMOVED IN A CASUALTY DEPARTMENT . . . *Professor T. F. Hewer* 14

POST-PARTUM NECROSIS OF THE PITUITARY *Clinical Pathological Conference* . . . 16

NEWS FROM SOCIETIES . . . . . . . . 20

NOTES AND NEWS . . . . . . . . 20

APPOINTMENTS . . . . . . . . . 21

*PUBLISHED QUARTERLY*

*ANNUAL SUBSCRIPTION* 16/- *POST FREE*

PUBLISHED UNDER THE AUSPICES OF THE
BRISTOL MEDICO-CHIRURGICAL SOCIETY

### *Editorial Committee*

Dr. J. E. Cates, Department of Medicine, Bristol Royal Infirmary. *Editor.*
Dr. N. J. Brown, Southmead Hospital, Bristol. *Assistant Editor.*
Dr. J. Macrae, Ham Green Hospital.
Dr. R. C. Wofinden, Central Clinic, Bristol.
Mr. A. E. S. Roberts, Medical Library, University of Bristol. *Secretary.*

### *Local Correspondents*

*Bath* . . Mr. A. D. Bateman, 3 The Circus, Bath.
*Exeter* . . Dr. L. N. Jackson, Mount Jocelyn, Crediton, Devon.
*Gloucester* . Dr. R. F. Jarrett, 9 College Green, Gloucester.
*Plymouth* . Dr. C. R. Croft, Lexden, Hartley Av., Plymouth.
*Taunton* . Dr. M. McAlley, 1 The Crescent, Taunton.
*Torquay* . Dr. A. Robinson Thomas, 20 Devon Square, Newton Abbot, Devon.
*Truro* . . Dr. F. D. M. Hocking, St. Andrews, Perranporth, Cornwall.
*Yeovil* . . Mr. J. A. Vere Nicoll, Knapp House, Yeovil, Somerset.

### NOTICE TO CONTRIBUTORS

Original articles are invited, provided they have not been published elsewhere. Notes of forthcoming events, meetings held, degrees conferred, staff appointments, and other local news are welcomed. The editorial committee reserves the right to make literary corrections.

Illustrations should always be supplied ready for reproduction. All re-touchings or other operations required will be charged to the author. Line drawings should be drawn in Indian ink. We regret that we must ask authors to pay for making blocks, if this is necessary.

J. W. ARROWSMITH LTD., WINTERSTOKE ROAD,
BRISTOL.

### *1959 Index to Vol. 74*

Acute Intestinal Obstruction, A Review of—P. J. W. Monks, 31

Alcock, N. S.—Recent Work on the Demyelinating Diseases, 17

Alveolar Cell Carcinoma of Lung—a Clinical Pathological Conference, 70

Animal Lung as Oxygenator in a Heart-Lung Machine, Use of an, The—M. G. Wilson and others, 90

Aplastic Anaemia with Post-Transfusional Haemosiderosis—a Clinical Pathological Conference, 47

Baby Farming in England.—Philip Evans, 83

Cardiac Surgery, Recent Developments in.—R. E. Horton, 6

Clinical Pathological Conferences:
—Alveolar Cell Carcinoma of Lung, 70
—Aplastic Anaemia with Post-Transfusional Haemosiderosis, 47
—Herpes Zoster with Chicken-pox and Lymphosarcoma, 22

Darling, A. I.—Decay of the Teeth, 53

Decay of the Teeth.—A. I. Darling, 53

Flavell Matts, S. G.—Prolonged Coma in Barbiturate Poisoning, 20

Hazell, Hanbury.—The Elimination of Piltdown Man, 1

Herpes Zoster with Chicken-pox and Lymphasarcomal—a Clinical Pathological Conference, 22

Horton, R. E.—Recent Developments in Cardiac Surgery, 6

Lung Cancer.—A Symposium.—J. E. G. Pearson, 61

Macrae, James.—Transporting Severely Paralysed Patients, 45

Monks, P. J. W.—A Review of Acute Intestinal Obstruction, 31

Pearson, J. E. G.—Lung Cancer—A Symposium, 61

Phillips, Calbert I.—Squint, 40

Piltdown Man, The Elimination of—Hanbury Hazell, 1

Prolonged Coma in Barbiturate Poisoning.—S. G. Flavell Matts, 20

Recent Work on the Demyelinating Diseases.—N. S. Alcock, 17

Squint.—Calbert I. Phillips, 40

Transporting Severely Paralysed Patients.—James Macrae, 45

Wilson, M. G. and others.—The use of an Animal Lung as Oxygenator in a Heart-Lung Machine, 90

